# Supplementary material for: A dataset for the study of identity at scale: Annual Prevalence of American Twitter Users with specified Token in their Profile Bio 2015–2020
Source: PLoS One. 2021 Nov 18;16(11):e0260185. doi: 10.1371/journal.pone.0260185 (PMC8601548; doi:10.1371/journal.pone.0260185)
Supplement: S1 File — (PDF) [file pone.0260185.s001.pdf]

Supplemental Material for

Annual Prevalence of American Twitter Users with specified Token in their Profile Bio 2015-2020: A Dataset for the Study of Identity at Scale

Jason Jeffrey Jones

https://orcid.org/0000-0002-4140-0268

Department of Sociology and Institute for Advanced Computational Science, Stony Brook University, Stony Brook, NY, United States of America

Token Prevalence Distribution in the Cross-Sectional Sample

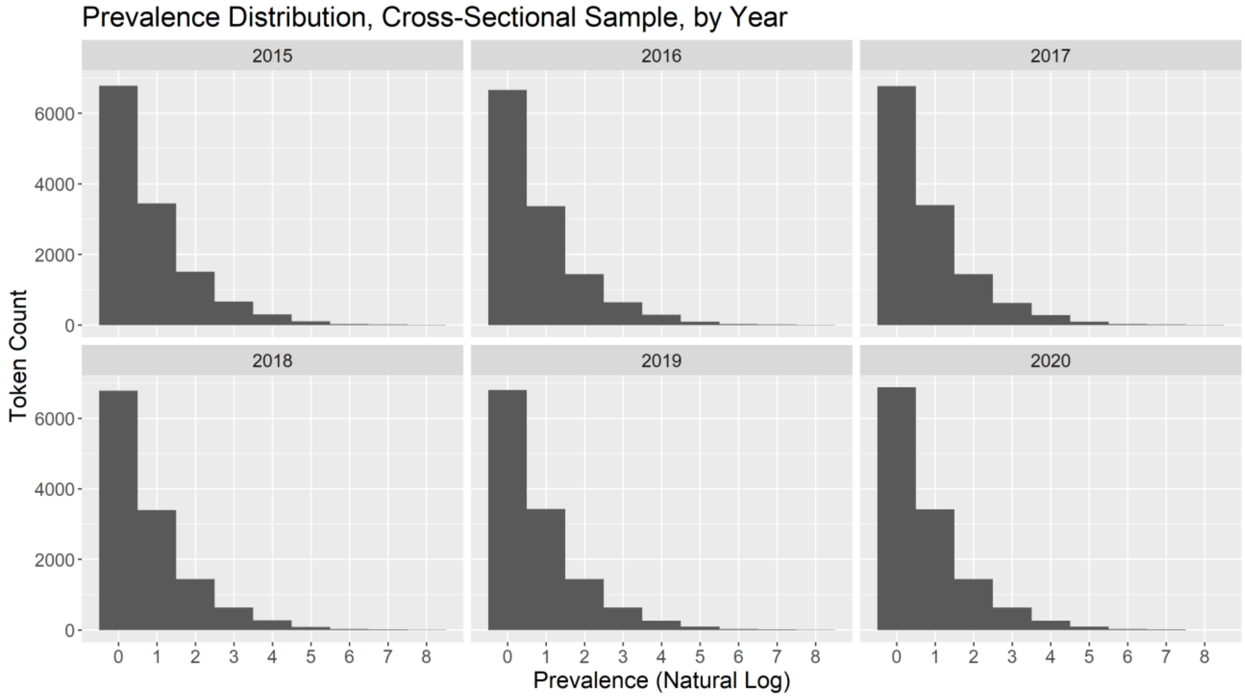

Token prevalence distributions per year for the cross-sectional sample. Note that the x-axis is on a log scale. There are a small number of high-prevalence tokens and large numbers of low-prevalence tokens. This accords with general expectations of word usage.

Best Distribution to Describe Token Prevalence

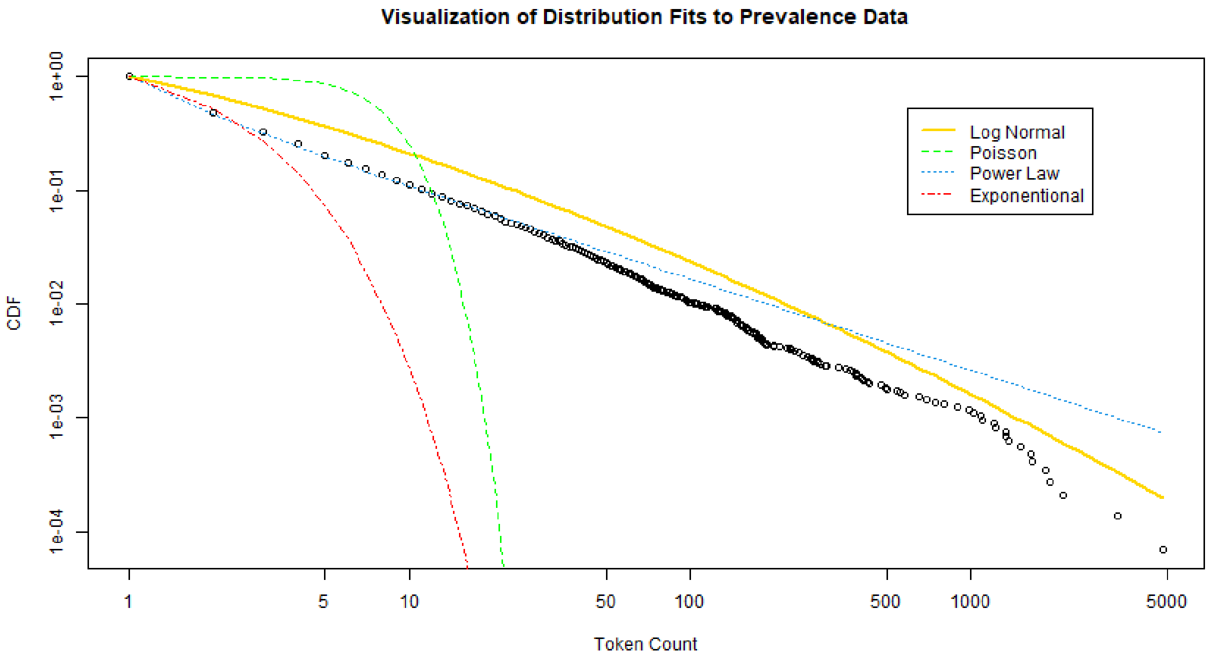

The Log Normal distribution provides the best fit to the distribution of Token Prevalence.

Tokens Excluded from Tables

Punctuation, single digits, a small set of stop word tokens (e.g. “at,” “be,” “on”) and a small set of URL tokens (e.g. “http,” “com”) were excluded from results reported in Tables. Full information regarding these tokens appears in the dataset. One can run the same analyses without filtering using the replication code provided.

Dataset and Code are Publicly Available

The dataset itself is publicly available at https://osf.io/guah5/. Code to replicate the analysis presented in the manuscript and Supplemental Material is publicly available at https://osf.io/rwxec/.
