# Supplementary material for: A dataset for the study of identity at scale: Annual Prevalence of American Twitter Users with specified Token in their Profile Bio 2015–2020
Source: PLoS One. 2021 Nov 18;16(11):e0260185. doi: 10.1371/journal.pone.0260185 (PMC8601548; doi:10.1371/journal.pone.0260185)
Supplement: Attachment — Submitted filename: Response to Reviewers.docx [file pone.0260185.s002.docx]

October 29, 2021

Dear Editors and Reviewers,

I thank you for your comments on my manuscript (PONE-D-21-22972) titled *A Dataset for the Study of Identity at Scale: Annual Prevalence of American Twitter Users with specified Token in their Profile Bio 2015-2020*.

In response to some points, I have revised the paper. On other points I respond here in this letter. Here are points made in review and my responses:

*Journal Requirements:*

*When submitting your revision, we need you to address these additional requirements.*

*1. Please ensure that your manuscript meets PLOS ONE's style requirements, including those for file naming. The PLOS ONE style templates can be found at*

*https://journals.plos.org/plosone/s/file?id=wjVg/PLOSOne_formatting_sample_main_body.pdf and*

*https://journals.plos.org/plosone/s/file?id=ba62/PLOSOne_formatting_sample_title_authors_affiliations.pdf*

I have made edits to ensure the manuscript meets PLOS ONE's style requirements. These include reordering the manuscript and revising headings to conform to PLOS ONE style.

*2. We note that the grant information you provided in the ‘Funding Information’ and ‘Financial Disclosure’ sections do not match.*

*When you resubmit, please ensure that you provide the correct grant numbers for the awards you received for your study in the ‘Funding Information’ section*

The following statement best describes the grant funding of this research. This statement should be included wherever the journal deems it to be necessary.

This material is based upon work supported by the National Science Foundation under grant IIS-1927227. The author thanks Stony Brook Research Computing and Cyberinfrastructure, and the Institute for Advanced Computational Science at Stony Brook University for access to the high-performance SeaWulf computing system, which was made possible by a $1.4M National Science Foundation grant (#1531492).

*3. Please remove your figures from within your manuscript file, leaving only the individual TIFF/EPS image files, uploaded separately. These will be automatically included in the reviewers’ PDF*

I have removed the figures from the manuscript file and uploaded the image files separately as requested.

*4. We note that Figure 1 in your submission contain copyrighted images. All PLOS content is published under the Creative Commons Attribution License (CC BY 4.0), which means that the manuscript, images, and Supporting Information files will be freely available online, and any third party is permitted to access, download, copy, distribute, and use these materials in any way, even commercially, with proper attribution. For more information, see our copyright guidelines: http://journals.plos.org/plosone/s/licenses-and-copyright.*

*We require you to either (1) present written permission from the copyright holder to publish these figures specifically under the CC BY 4.0 license, or (2) remove the figures from your submission:*

*a. You may seek permission from the original copyright holder of Figure 1 to publish the content specifically under the CC BY 4.0 license.*

*We recommend that you contact the original copyright holder with the Content Permission Form (http://journals.plos.org/plosone/s/file?id=7c09/content-permission-form.pdf) and the following text:*

*“I request permission for the open-access journal PLOS ONE to publish XXX under the Creative Commons Attribution License (CCAL) CC BY 4.0 (http://creativecommons.org/licenses/by/4.0/). Please be aware that this license allows unrestricted use and distribution, even commercially, by third parties. Please reply and provide explicit written permission to publish XXX under a CC BY license and complete the attached form.”*

*Please upload the completed Content Permission Form or other proof of granted permissions as an "Other" file with your submission.*

*In the figure caption of the copyrighted figure, please include the following text: “Reprinted from [ref] under a CC BY license, with permission from [name of publisher], original copyright [original copyright year].”*

*b. If you are unable to obtain permission from the original copyright holder to publish these figures under the CC BY 4.0 license or if the copyright holder’s requirements are incompatible with the CC BY 4.0 license, please either i) remove the figure or ii) supply a replacement figure that complies with the CC BY 4.0 license. Please check copyright information on all replacement figures and update the figure caption with source information. If applicable, please specify in the figure caption text when a figure is similar but not identical to the original image and is therefore for illustrative purposes only.*

I have removed Figure 1 from the manuscript. It was included for illustration and not necessary for understanding the manuscript.

*5. Please review your reference list to ensure that it is complete and correct. If you have cited papers that have been retracted, please include the rationale for doing so in the manuscript text, or remove these references and replace them with relevant current references. Any changes to the reference list should be mentioned in the rebuttal letter that accompanies your revised manuscript. If you need to cite a retracted article, indicate the article’s retracted status in the References list and also include a citation and full reference for the retraction notice.*

I have reviewed the reference list to ensure it is complete and correct. To my knowledge, the article does not and did not cite any retracted work. Please inform me if that is not accurate.

*Reviewer #1*

*The paper presents a valuable contribution regarding the use of online data to study the evolution of social trends in the US over the 2015-2020 time frame. The author precisely describes the methodology used to compile the dataset, the metrics developed to analyze it, and shows a few research avenues that could be investigated through this data.*

*While the language used by the author is clear and precise, the structure of the article could sometimes be clarified. Some methodological elements are defined several times over the article (such as the calculation of prevalence, page 7 and again page 14). The section on* *"Longitudinal Online Profile Sampling" appears after the presentation of the dataset. The paper could be improved with a more rigorous separation of sections such as data collection, metrics, online tool, examples ...*

I have re-ordered and revised headings in response to this feedback and the request to conform to journal formatting. Specifically, "Longitudinal Online Profile Sampling" section appears in the Materials and Methods section before the presentation of the dataset (in the Results section) as the reviewer requested. Also, the calculation of prevalence is defined only once in this revision.

*The simple tokenisation approach described in the article (separation over word boundaries) seems to provide interesting results. However, some classic linguistic methods such as lemmatization could be investigated to study not only one given form of a term ("kitty", "kitties", "kitten") but all the form of a given term ("kitt-" or even "cat-"). Such process could be an interesting addition to the dataset.*

I thank the reviewer for the suggestion, but argue this form of analysis does not belong in this manuscript. Modern approaches to Natural Language Processing often skip lemmatization. Lemmatization is useful when one has limited text data, and it is assumed different linguistic forms necessarily relate to the same semantic content. But in my application, language is plentiful, and I am specifically interested in the exact words users choose to represent themselves.

*Finally, I have to point out that the name and URL of the online tool infringe the anonymity of the author. When submitting this kind of paper through a peer review process, the name of the tool should be anonymized and the URL should only be integrated in the final version.*

The name of the tool and the URL includes my full name intentionally. Scientists should demand and receive more credit for their work. This journal does not require blind review. Even in venues that promise blind review, that promise is rarely delivered upon. A Google search on the title or any unique phrasing would reveal the identity of the author of this work (me), and that is something I cannot control.

*Reviewer #2:*

*I have read and reviewed ‘A Dataset for the Study of Identity at Scale: Annual Prevalence of American Twitter. Users with specified Token in their Profile Bio 2015-2020’. This article provides information about a new method and data set to study self-reported self-concept via the Twitter bio, and trends therein. The article is written very well and describes an interesting and relevant method for analyzing trends in the way people chose to present themselves.*

*And in this last sentence lies my only major criticism of the present article: it strikes me as purely descriptive, and would benefit from a little more background with regard to the theme of self-concept. The references the author gives on this topic seem incomplete. I would personally recommend a bit more information about what type of self-representation a Twitter bio would constitute on the basis of literature on the topic of self-categorization and generally the psychology of identity. I would suggest the literature listed (incompletely!) below. Without more information about the psychology of how and why people chose to represent themselves in the way they do, the analysis is rather superficial and only describes trends (‘winners and losers’), in my opinion. To add any sort of meaning to those trends, I think more background is needed.*

The reviewer and I have a difference of opinion. These suggested critiques are in my opinion strengths of the manuscript. “Purely descriptive” is used a pejorative by the reviewer, but in my opinion descriptive work is sorely needed, unduly maligned, and deeply underrepresented in the study of human behavior and especially identity.

“What is the self?” is a fascinating question, but one that has been written about elsewhere at length. I don’t intend to address this question directly in this manuscript. This manuscript’s primary purpose is to introduce a dataset. A survey of ideas of the self-concept could only be sprawling or insufficient in that context. More crucially, it would bias readers’ percepetion of the dataset and the method as associated with a particular theory or set of theories. This would be disingenuous and misleading, because the current effort is data-driven and purposely descriptive in nature.

*This would also provide more insight into questions about to what degree the medium, Twitter, influences the way in which people choose to represent themselves.*

It is not my intention to address how the medium (Twitter) influences self-representation. Twitter is simply the only source of personally expressed identity data for millions of people across time and space. I leave it to other researchers to seek and explain cross-media effects.

*To be perfectly clear: my remarks are only valid if the author intended to not only introduce the dataset, and the data collection method, but also describe how this data can be analyzed. If not, than the article is concise and short description of said aspects.*

*In sum: the author describes an interesting and potentially promising method for collecting self-reported data on identity. The article is well written and the used methods are sound. As a reviewer I feel however, that a more complete discussion of the relevant literature on self-identity, self-categorization and self-representation would help to better appreciate the value of the collected data.*

Here the reviewer and I agree. There is much more to be said about self-identity, self-categorization and self-representation than is in this manuscript. I read the reviewer’s included citations with interest. My aim being a concise description of methods and data, however, I choose to not delve into the history of the many conceptualizations of the self. I hope the editor and reviewers agree this is a fine choice for this manuscript.

Thank you for your time and effort in considering this manuscript.

Please address all correspondence concerning this manuscript to me: [jason.j.jones@stonybrook,edu](mailto:jason.j.jones@stonybrook,edu).

Sincerely,

Dr. Jason J. Jones

Associate Professor

Dept. of Sociology and Institute for Advanced Computational Science

Stony Brook University
